# Impact of angiotensin receptor–neprilysin inhibition on vascular function in heart failure with reduced ejection fraction: A pilot study

**DOI:** 10.14814/phy2.15209

**Published:** 2022-03-05

**Authors:** Sangeetha Nathaniel, Shane McGinty, Melissa A. H. Witman, David G. Edwards, William B. Farquhar, Vinay Hosmane, Megan M. Wenner

**Affiliations:** ^1^ Department of Kinesiology and Applied Physiology University of Delaware Newark Delaware USA; ^2^ Hosmane Cardiology and Section of Cardiology Christiana Care Healthcare System Newark Delaware USA

**Keywords:** ARNi, arterial stiffness, endothelial function, flow‐mediated dilation, pulse wave velocity

## Abstract

The mechanisms for the benefits of Angiotensin Receptor Neprilysin Inhibition (ARNi) in heart failure patients with reduced ejection fraction (HFrEF) are likely beyond blood pressure reduction. Measures of vascular function such as arterial stiffness and endothelial function are strong prognostic markers of cardiovascular outcomes in HFrEF, yet the impact of ARNi on vascular health remains to be explored. We hypothesized that arterial stiffness and endothelial function would improve after 12 weeks of ARNi in HFrEF. We tested 10 stable HFrEF patients at baseline and following 12 weeks of ARNi [64 ± 9 years, Men/Women: 9/1, left ventricular ejection fraction (EF): 28 ± 6%] as well as 10 stable HFrEF patients that remained on conventional treatment (CON: 60 ± 7 years, Men/Women: 6/4, EF: 31 ± 5%; all *p* = NS). Arterial stiffness was assessed via carotid‐femoral pulse wave velocity (PWV) and endothelial function was assessed via brachial artery flow‐mediated dilation (FMD). PWV decreased after 12 weeks of ARNi (9.0 ± 2.1 vs. 7.1 ± 1.2 m/s; *p* < 0.01) but not in CON (7.0 ± 2.4 vs. 7.5 ± 2.3 m/s; *p* = 0.35), an effect that remained when controlling for reductions in mean arterial pressure (*p* < 0.01). FMD increased after 12 weeks of ARNi (2.2 ± 1.9 vs. 5.5 ± 2.1%; *p* < 0.001) but not in CON (4.8 ± 3.8 vs. 5.4 ± 3.4%; *p* = 0.34). Baseline PWV (*p* = 0.06) and FMD (*p* = 0.07) were not different between groups. These preliminary data suggest that 12 weeks of ARNi therapy may reduce arterial stiffness and improve endothelial function in HFrEF. Thus, the findings from this pilot study suggest that the benefits of ARNi are beyond blood pressure reduction and include improvements in vascular function.

## Graphical abstract

ARNi may improve vascular function in HFrEF when compared to conventional treatment.

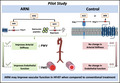

**New & Noteworthy**

Twelve weeks of ARNi therapy may reduce arterial stiffness (assessed by carotid‐femoral PWV) and improve endothelial function (assessed by brachial artery FMD) in HFrEF when compared to conventional treatment. Improvement in vascular function may be a physiological mechanism for the clinical benefit seen with ARNi in HFrEF. Moreover, these pleiotropic benefits of ARNi beyond BP lowering may be vital for the treatment of HFrEF and possibly other cardiovascular diseases.

## 1 INTRODUCTION

Angiotensin Receptor–Neprilysin Inhibitor (ARNi) is the first of a class of dual combination drugs that block angiotensin II receptors and inhibits neprilysin (Maddox et al., 2021). ARNi was approved for use in patients with heart failure with reduced ejection fraction (HFrEF) classified as New York Heart Association (NYHA) functional class II‐IV (Maddox et al., 2021). In clinical trials, ARNi effectively reduced morbidity and mortality in HFrEF compared to traditional treatment (Braunwald, 2015; McMurray et al., 2014). However, the greater mortality reduction could not be attributed solely to reductions in blood pressure (BP) as observed in previous trials (Collaboration, 2002). Thus, the clinical benefits noted with ARNi likely extend beyond BP reduction; however, other mechanism(s) for improved benefits are unclear. Having a complete understanding of the cardiovascular (CV) physiology of ARNi is crucial as it may be key for future therapies in HFrEF and other CV diseases. ARNi, by neprilysin inhibition, increases endogenous natriuretic peptides (NP), augmenting their cardiovascular pleiotropic effects (Forte et al., 2019). As vascular dysfunction plays a vital role in the initiation and clinical progression of heart failure (Marti et al., 2012), improvements in vascular function have been an innately attractive potential physiological mechanism for the benefits seen with this new pharmacotherapy.

Vascular stiffening is pathological in several CV diseases, including HFrEF (Patrianakos et al., 2009). Increased large artery stiffness impairs optimal ventricular‐vascular interaction by increasing left ventricular (LV) afterload (Paglia et al., 2014) and impeding effective stroke volume. In HFrEF, aortic stiffness contributes to cardiac remodeling and myocardial fibrosis (Puntmann et al., 2014), further deteriorating LV function and accelerating the progression of heart failure pathophysiology. Increased carotid‐femoral pulse wave velocity (PWV), a measure of central arterial stiffness, is associated with LV dysfunction (Weber et al., 2008). Furthermore, PWV is an independent predictor of exercise tolerance (Bonapace et al., 2003) and cardiac mortality or hospitalizations (Bonapace et al., 2013) in patients with HFrEF. Interventions aimed at attenuation of PWV are associated with improved survival in end‐stage renal disease (Guerin et al., 2001) and reduction in pulsatile afterload improves functional capacity in patients with HFrEF (Wohlfahrt et al., 2017). Previous studies in animals have shown that ARNi ameliorates aortic fibrosis in chronic kidney disease animal models (Suematsu et al., 2018). In HFrEF animal models, ARNi improved nitric oxide (NO) bioavailability, aortic vasorelaxation responses, and vascular compliance (Trivedi et al., 2018). In humans, ARNi showed a trend of greater reduction in PWV in elderly patients with systolic hypertension (Williams et al., 2017). However, to date, the impact of ARNi on PWV in humans with HFrEF is unknown. Thus, improvements in PWV may be a potential mechanism for the benefits seen with ARNi in HFrEF, and it is imperative to evaluate the impact of ARNi on PWV in HFrEF.

Peripheral vascular dysfunction, particularly endothelial dysfunction, is a hallmark finding in HFrEF (Drexler et al., 1992; Katz et al., 1992; Witman et al., 2015). Endothelial function measured by flow‐mediated dilation (FMD) of the brachial artery correlates with heart failure severity (Meyer et al., 2005). Additionally, in patients with HFrEF, endothelial dysfunction is an independent predictor of hospitalizations, cardiac transplantation, and mortality (Fischer et al., 2005; Shechter et al., 2009). Improvements in endothelial function have been shown to enhance outcomes in HFrEF (Hambrecht et al., 1998). Recently, a study evaluated brachial artery FMD in eleven HFrEF patients at baseline and 1, 2, and 3 months after ARNi initiation. ARNi improved FMD after one month of treatment, which persisted at two and three months (Bunsawat et al., 2020). These preliminary findings are intriguing and open a new paradigm of a possible mechanism for clinical benefits seen with ARNi in HFrEF. Nevertheless, the lack of a control arm in this study to evaluate the natural progression of endothelial function in HFrEF on traditional goal‐directed medical therapy makes it challenging to assess the benefits of ARNi over conventional treatment.

There is still a lack of complete understanding of the impact of ARNi on global vascular function in humans. The benefits of ARNi over goal‐directed medical therapy in HFrEF on endothelial function still need to be established. Furthermore, the impact of ARNi on other parameters of vascular function has not been assessed in HFrEF and needs to be explored, especially the impact of ARNi on arterial stiffness in HFrEF. Accordingly, the purpose of this pilot study was to test the hypothesis that 12 weeks of ARNi therapy in patients with HFrEF would reduce central arterial stiffness, assessed by carotid‐femoral PWV, and improve endothelial function, assessed by brachial artery FMD when compared to conventional treatment.

## 2 METHODS

All procedures were approved by the University of Delaware Institutional Review Board, which were in accordance with the principles outlined in the Declaration of Helsinki. Informed consent was obtained from all participants. We recruited stable HFrEF participants (*N* = 20) with an ejection fraction of ≤35% and NYHA functional Class II‐III symptoms from cardiology or primary care offices in the greater Newark, DE area. Ten participants were starting on ARNi (Sacubitril/Valsartan) under the direction of their physician, whereas 10 participants (control; CON) remained on conventional therapy [Angiotensin converting enzyme inhibitors (ACEi)/angiotensin receptor blockers (ARB)]. Resting BP, height, and weight was measured at study enrollment. Exclusion criteria included symptomatic hypotension [systolic BP (SBP) <100 mm Hg and diastolic BP (DBP) <60 mm Hg], severe chronic kidney disease (CKD stage 4 or 5), history of angioedema or previous side effects from ACEi/ARBs and frequent severe cardiac arrhythmias (ventricular tachycardia or ventricular fibrillation) or hospitalizations in the last 1 month before inclusion in the study. Only those with stable HFrEF and no other unstable disease symptoms in the past month were accepted into the study.

### 2.1 Study design

Measurement of arterial stiffness (carotid‐femoral pulse wave velocity, PWV) and endothelial function (brachial artery flow‐mediated dilation, FMD) occurred at baseline and following 12 weeks of therapy. For the ARNi group, the baseline visit while on conventional treatment, and 3–7 days before initiating ARNi. Control subjects that were continuing on conventional therapy for HFrEF were tested at a time of their convenience. Each participant worked with their cardiologist to titrate their medications on an individual basis. ARNi was initially started at the lowest dose (24/26 mg mg) and was titrated by the participant's cardiologists on an individual basis every 4 weeks to the maximal dose (97/103 mg), as tolerated. Participants returned to the laboratory for follow‐up assessment of arterial stiffness and endothelial function after 12 weeks. Participants were given an accelerometer (ActiGraph, Florida, USA; CE) to wear for 1 week prior to each testing session (baseline and 12 wk follow‐up) to assess any changes in sedentary time levels during the study timeframe. The accelerometer was worn on the hip at all times (except when bathing and sleeping). The Kansas City Cardiomyopathy Questionnaire (KCCQ‐12), which is a reliable and valid self‐administered questionnaire for measuring the disease‐specific quality of life in heart failure (Spertus & Jones, 2015), was completed at baseline and 12‐week follow‐up visits.

### 2.2 Experimental protocol & measurements

Participants reported to the laboratory after an overnight fast and were asked to avoid caffeine and alcohol for 12 h prior and exercise 24 h prior to the experimental visit. All experimental visits were performed in a temperature‐controlled environment. After emptying their bladder, height and weight were measured. Participants lay in the supine position for 15 min, and peripheral brachial SBP and DBP (Pickering et al., 2005) were measured in triplicate using an automated unit (GE Medical Systems Dinamap Dash 2000, Milwaukee, WI). A blood sample was obtained for a comprehensive metabolic profile and complete blood count.

#### 2.2.1 Arterial stiffness

In the supine position, carotid‐femoral PWV was measured using the SphygmoCor XCEL system (AtCor Medical). A BP cuff was placed on the upper thigh. A high‐fidelity strain gauge transducer (Millar Instruments, Houston, TX, USA) was placed on the carotid artery. From the carotid and femoral waveforms, pulse transit time was determined as the time delay between the base of carotid and femoral waveforms. The distance between the two measurement sites (carotid and femoral) was measured from the carotid artery to the sternal notch and the sternal notch to the tip of the BP cuff on the upper thigh using a cloth tape. PWV was calculated within the system by dividing the distance between two measurement sites by the pulse transit time. ∆PWV was also calculated to determine the change in PWV from baseline to 12 weeks follow‐up (i.e., 12 weeks post‐initiation of ARNi minus baseline) in both groups.

#### 2.2.2 Endothelial function

Conduit artery endothelial function was assessed using FMD of the brachial artery in accordance with current recommendations (Thijssen et al., 2019) and previously described by our group (DuPont et al., 2013; Matthews et al., 2015; Wenner et al., 2017). Briefly, participants were tested in a supine position, with the left arm supported at the heart level, legs uncrossed after at least 10 min of quiet rest. An occlusion BP cuff (Hokanson Rapid Cuff Inflator; Hokanson, Bellevue, WA) was placed distal to the olecranon process. A linear phased‐array ultrasound transducer (GE Logiq e, Healthcare, Wauwatosa, WI) was used to acquire longitudinal images of the brachial artery and Doppler blood velocity continuously throughout the protocol. Baseline images were recorded for 1 min. Then, the cuff was rapidly inflated to supra‐systolic pressures (>200 mm Hg) for 5 min. Following deflation, data continued to be acquired for an additional 2 min. The diameter of the brachial artery was determined using automated edge‐detection software (Quipu). FMD is expressed as a percentage change from baseline diameter to peak diameter reached following deflation (Harris et al., 2010). Shear rate was calculated from Doppler data as 8 × Velocity × vessel diameter^−1^. The shear stimulus was calculated as the area under the curve of the shear rate (SR_AUC_) profile from cuff deflation to peak diameter. Brachial artery FMD is sensitive to detect differences in endothelial function in HFrEF patients (Cai & Harrison, 2000; Fischer et al., 2005; Shechter et al., 2009). All FMDs were performed by a single investigator (SN) to keep inter‐observer variability low, and all acquired images were blinded and analyzed offline. Previously, our laboratory has shown consistent reproducibility in FMD measurements (DuPont et al., 2013). ∆FMD was also calculated to determine the change in FMD from baseline to 12 weeks follow‐up (i.e., 12 weeks post‐initiation of ARNi minus baseline) in both groups.

### 2.3 Statistical analysis

An a priori power analysis was conducted using G*Power software to determine sample size. Based on preliminary data collected in our laboratory for PWV and a calculated effect size of 0.55 indicating 10 participants in each group would provide adequate power (0.80 1 − β err prob), with α ≤ 0.05. Subject demographics were compared between treatment groups using an independent *t*‐test. A 2 × 2 Analysis of variance (ANOVA) was used to determine differences in vascular function measures and post‐hoc LSD tests were conducted to evaluate pairwise differences among the means. ∆PWV and ∆FMD were compared between two groups using *t*‐test. All data are presented as mean ±standard deviation (SD). All data were analyzed using SPSS v24.0 (Chicago, IL, USA).

## 3 RESULTS

### 3.1 Subject characteristics

The demographic and clinical characteristics of both groups are shown in Tables 1 and 2. There was no significant difference in age, sex, and other clinical and biochemical parameters between the two groups (Table 1). All participants were on goal‐directed medical therapy for HFrEF, including statins and β‐blockers, and all participants were on ACEi/ARBs at baseline (Table 2). There were no significant changes in pharmacotherapy or device therapy during the course of follow‐up of all participants. There were also no hospitalizations during this follow‐up period.

**TABLE 1:** Subject characteristics and blood chemistry at study initiation

	ARNi (*N* = 10)	Control (*N* = 10)
Subject characteristics		
Age (years)	64 ± 9	60 ± 7
Men/women	9/1	6/4
BMI (Kg/m^2^)	31.1 ± 5.6	31.0 ± 6.0
EF (%)	28 ± 6	31 ± 5
NYHA II/III	4/6	5/5
Ischemic cardiomyopathy	4/10	6/10
Nonischemic cardiomyopathy	6/10	4/10
Hypertension	9/10	7/10
Dyslipidemia	9/10	9/10
Chronic kidney disease	4/10	3/10
Diabetes	2/10	2/10
Blood values at baseline		
Total cholesterol (mg/dl)	131 ± 31	137 ± 38
HDL (mg/dl)	40 ± 11	44 ± 14
Triglycerides (mg/dl)	100 ± 39	109 ± 67
LDL (mg/dl)	71 ± 25	74 ± 26
BUN (mg/dl)	19 ± 7	19 ± 8
Creatinine (mg/dl)	1.2 ± 0.4	1.3 ± 0.7
Glucose (mg/dl)	129 ± 109	102 ± 27
Hemoglobin (g/dl)	13 ± 2	13 ± 1
Hematocrit (%)	39 ± 6	39 ± 3

Note

Values are *n* or mean ± SD.

Abbreviations: BMI, body mass index; BUN, blood urea nitrogen; EF, ejection fraction; HDL, high‐density lipoprotein; LDL, low‐density lipoprotein; NYHA, New York heart association.

**TABLE 2:** Medication use at study initiation

	ARNi (*N* = 10)	Control (*N* = 10)
β‐blocker	10/10	10/10
ACEi/ARB	10/10	10/10
Statin/Lipid lowering medications	10/10	10/10
Aldosterone antagonist	1/10	0/10
Hydralazine/Nitrates	1/10	1/10
Loop diuretics	4/10	4/10
Antiplatelet	10/10	10/10
Anticoagulant	5/10	2/10

Note

Values are *n*.

Abbreviations: ACEi, angiotensin‐converting‐enzyme inhibitors; ARB, angiotensin receptor blockers.

### 3.2 Peripheral blood pressure & heart rate

Peripheral SBP, DBP, mean arterial pressure (MAP), and heart rate (HR) in ARNi and CON groups are shown in Table 3. Baseline SBP was not statistically different between ARNi and CON (*p* = 0.10); SBP decreased after 12 weeks of ARNi (*p* = 0.01) with no change in CON (*p* = 0.09). Baseline DBP was greater in ARNi compared to CON (*p* = 0.01); DBP decreased after 12 weeks of ARNi (*p* = 0.04) with no change in CON (*p* = 0.36). Baseline MAP was greater in ARNi compared to CON (*p* = 0.02); 12 weeks of ARNi therapy improved MAP (*p* = 0.01) but there was no change in CON (*p* = 0.16). HR was similar at baseline between both groups and was unchanged after 12 weeks (all *p* = NS).

**TABLE 3:** Brachial blood pressure and heart rate in ARNi and control groups at baseline and 12 weeks follow‐up

	ARNi baseline	ARNi 12 weeks POST	Control baseline	Control 12 weeks POST	Statistics
SBP (mm Hg)	134 ± 19	118 ± 11^a^	120 ± 18	130 ± 31	Group: *p* = 0.92 Time: *p* = 0.47 Interaction: *p* < 0.01
DBP (mm Hg)	78 ± 7	71 ± 6^a^	69 ± 7^b^	71 ± 10	Group: *p* = 0.12 Time: *p* = 0.38 Interaction: *p* = 0.04
MAP (mm Hg)	96 ± 9	87 ± 7^a^	86 ± 9^b^	91 ± 16	Group: *p* = 0.46 Time: *p* = 0.39 Interaction: *p* = 0.01
HR (bpm)	62 ± 13	61 ± 10	61 ± 11	62 ± 9	Group: *p* = 0.98 Time: *p* = 0.96 Interaction: *p* = 0.54

Abbreviations: DBP, diastolic blood pressure; HR, heart rate; MAP, mean arterial pressure; SBP, systolic blood pressure.

^a^ *p* < 0.05 ARNi baseline versus 12 weeks POST.

^b^ *p* < 0.05 ARNi baseline versus Control Baseline.

### 3.3 Pulse wave velocity

**FIGURE 1:**
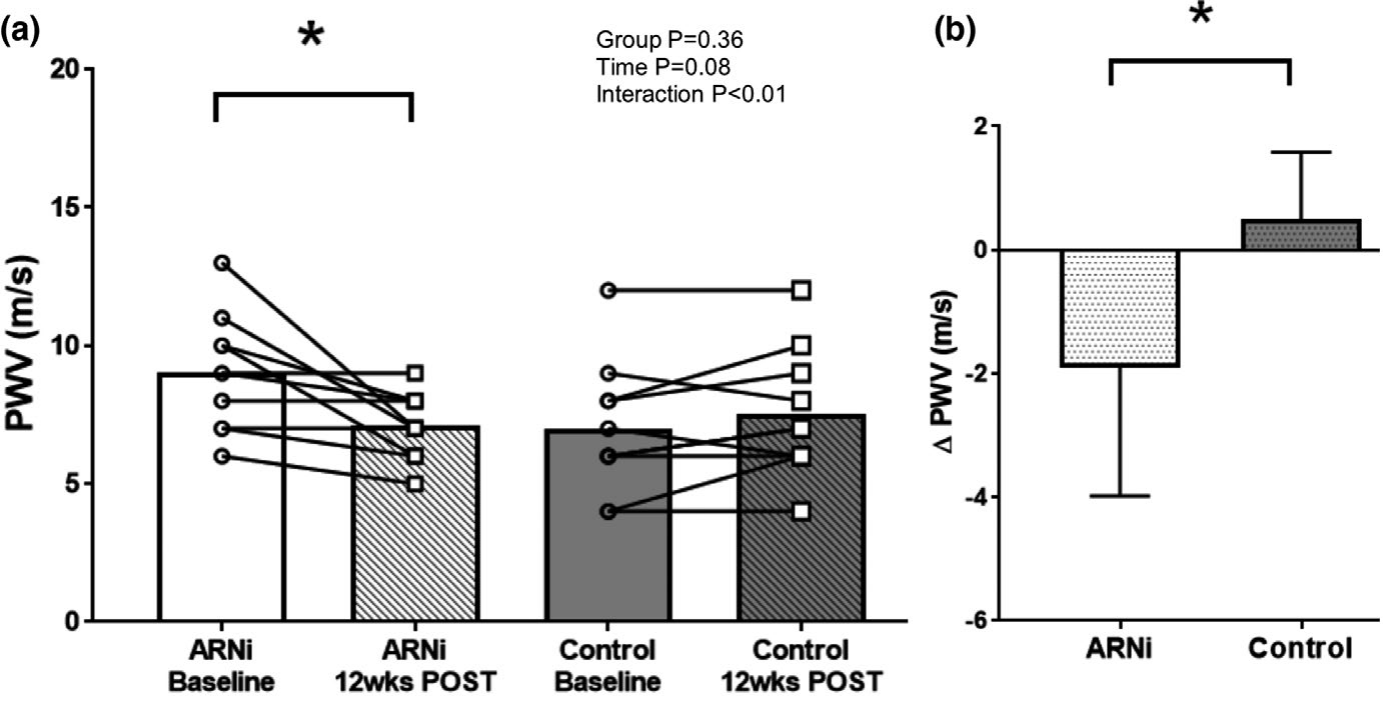
(a) Pulse Wave Velocity (PWV) in ARNi (*n* = 10; 9 men and 1 woman) and Control groups (*n* = 10; 6 men and 4 women) at baseline and 12 weeks follow‐up. A 2 × 2 Analysis of variance (ANOVA) was used to determine differences in vascular function measures and post‐hoc LSD tests were conducted to evaluate pairwise differences among the means. ∆PWV was compared between two groups using *t*‐test. PWV was similar between ARNi and Control at baseline; PWV improved after 12 weeks of ARNi and remained unchanged in Control. (b) ∆PWV in ARNi and Control groups. ∆PWV in ARNi was lower when compared to Control. *p < 0.01

As shown in Figure 1a, there was a significant group by drug interaction for PWV (ANOVA group *p* = 0.36; time *p* = 0.08; interaction *p* < 0.01). ARNi therapy reduced PWV over 12‐weeks (9.0 ± 2.1 vs. 7.1 ± 1.2 m/s; *p* < 0.01) whereas no change in PWV was observed in CON (7.0 ± 2.4 vs. 7.5 ± 2.3 m/s; *p* = 0.35). When PWV was analyzed with MAP as a covariate using one‐way analysis of covariance (ANCOVA), the impact of ARNi persisted (ARNi baseline vs. 12 weeks follow‐up: *p* < 0.01; ANCOVA group *p* = 0.09; time *p* = 0.09; interaction *p* = 0.02). Baseline PWV was not statistically different between ARNi and CON (*p* = 0.06). Figure 1b shows the ∆PWV within ARNi was greater compared to CON (−1.98 ± 2.09 m/s vs. 0.43 ± 1.15 m/s; *p* < 0.01).

### 3.4 Flow mediated dilation

**FIGURE 2:**
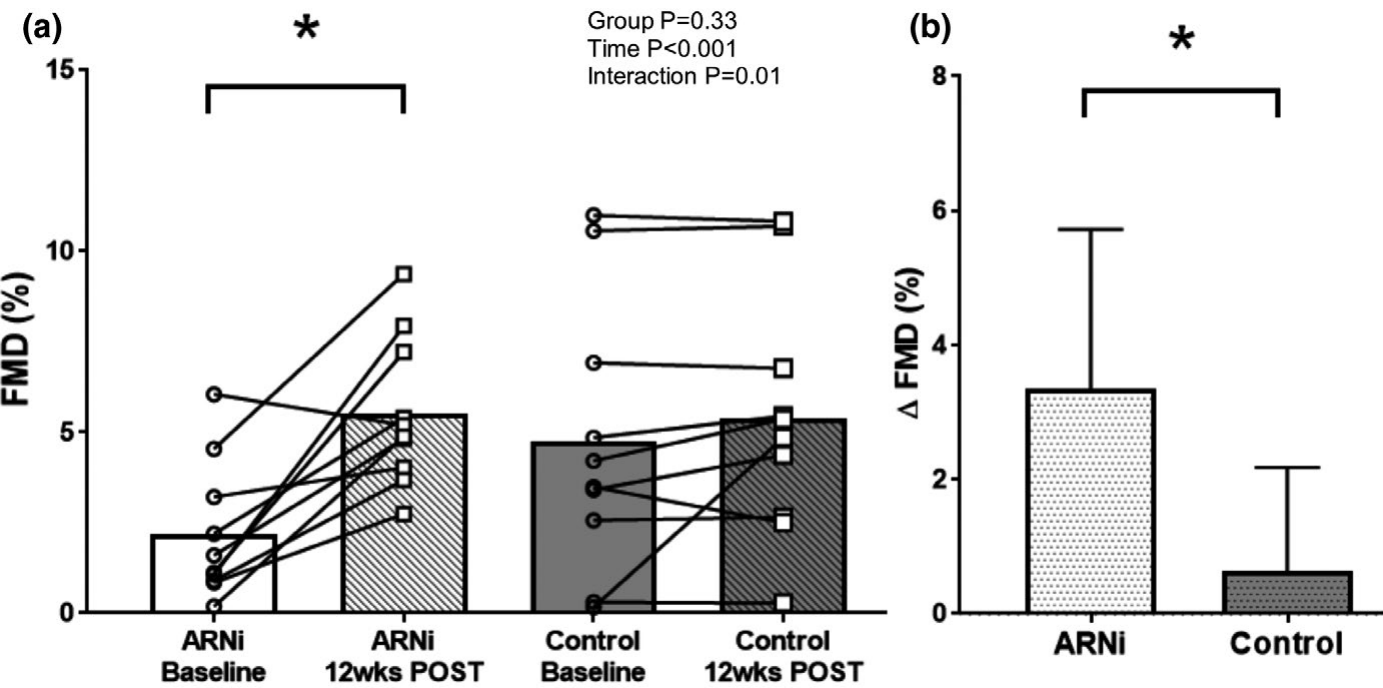
(a) Flow mediated dilation (FMD) in ARNi (*n* = 10; 9 men and 1 woman) and Control groups (*n* = 10; 6 men and 4 women) at baseline and 12 weeks follow‐up. A 2 × 2 Analysis of variance (ANOVA) was used to determine differences in vascular function measures and post‐hoc LSD tests were conducted to evaluate pairwise differences among the means. ∆FMD was compared between two groups using *t*‐test. FMD was similar between ARNi and Control at baseline; FMD improved after 12 weeks of ARNi and remained unchanged in Control. (b) ∆FMD in ARNi and Control groups. ∆FMD in ARNi was higher when compared to Control. *p < 0.001

As shown in Figure 2a, there was a significant group by drug interaction for FMD (ANOVA group *p* = 0.33; time *p* < 0.001, interaction *p* = 0.01). ARNi therapy increased FMD over 12‐weeks (2.2 ± 1.9 vs. 5.5 ± 2.1%; *p* < 0.001), whereas no change was noted in CON (4.8 ± 3.8 vs. 5.4 ± 3.4%; *p* = 0.34). When FMD was analyzed using ANCOVA with MAP as a covariate, the effect of ARNi persisted (ARNi baseline vs. 12 weeks follow‐up *p* < 0.001; ANCOVA group *p* = 0.35; time *p* < 0.01; interaction *p* = 0.02). Baseline FMD was not statistically different between ARNi and CON (*p* = 0.07). Figure 2b shows ∆FMD was greater in ARNi compared to CON (3.4 ± 2.4% vs. 0.6 ± 1.6%; *p* < 0.01). Baseline diameter of the brachial artery was similar between ARNi (5.7 ± 1.1 mm) and CON (5.3 ± 1.5 mm; *p* = 0.56); baseline diameter remained unchanged after 12 weeks of ARNi (6.1 ± 1.4 mm; *p* = 0.23) and also in CON (5.4 ± 1.2 mm; *p* = 0.72) (ANOVA group *p* = 0.35; time *p* = 0.27; interaction *p* = 0.54). Similarly, SR_AUC_ was similar between ARNi (11.5 ± 5.5 × 10^3^) and CON (16.6 ± 10.9 × 10^3^; *p* = 0.21); SR_AUC_ remained unchanged after 12 weeks of ARNi (11.8 ± 7.0 × 10^3^; *p* = 0.94) and also remained unchanged in CON (22.6 ± 11.8 × 10^3^; *p* = 0.10) (ANOVA group *p* = 0.03; time *p* = 0.22; interaction *p* = 0.26).

### 3.5 Sedentary time and Kansas City cardiomyopathy questionnaire

Sedentary time (h/day), as measured by an accelerometer, did not change in either ARNi (8.8 ± 1.5 vs. 8.5 ± 2.4 h/day) or CON (10.3 ± 2.6 vs. 9.2 ± 2.9 h/day) during the 12‐weeks (ANOVA group *p* = 0.24; time *p* = 0.28; interaction *p* = 0.57). KCCQ‐12 scores also did not change in ARNi (72 ± 24 vs. 80 ± 20) or CON (77 ± 23 vs. 79 ± 23) during the 12‐weeks (ANOVA group *p* = 0.82; time *p* = 0.07; interaction *p* = 0.37).

## 4 DISCUSSION

The main novel findings of this pilot study suggest that 12 weeks of ARNi therapy may reduce arterial stiffness and improve endothelial function in patients with HFrEF. These improvements in vascular function persisted even when controlling for reductions in BP that occur with ARNi. The current preliminary data adds to prior clinical findings to show the benefits of ARNi extend beyond BP reduction and may represent pleiotropic CV benefits of ARNi.

### 4.1 ARNi and arterial stiffness

To our knowledge, we are the first to demonstrate that ARNi improves arterial stiffness as measured by PWV in HFrEF patients after 12 weeks. From previous studies, there is conflicting evidence concerning the evaluation of arterial stiffness in HFrEF (Mitchell et al., 2001; Regnault et al., 2014). These conflicting data may be due to methodological differences in the assessment of arterial stiffness. Using characteristic aortic impedance, a recent randomized trial did not show a significant change in arterial stiffness in HFrEF after 12 weeks of ARNi therapy (Desai et al., 2019). However, our data show a significant improvement in carotid‐femoral PWV with ARNi in HFrEF. The reason for this discrepancy is not entirely clear, but may be due to the inherent differences in the population studied; the prior study included ~80% of patients with NYHA functional Class I‐II, whereas patients in our study had NYHA functional class II‐III symptoms. In a previous trial on elderly patients with systolic hypertension, ARNi showed a trend of greater reduction in PWV, especially in the subgroup of patients who were in the upper quartile based on baseline PWV (Williams et al., 2017). Hypertensive patients in the upper quartile based on baseline PWV reduced PWV with ARNi by 1.8 m/s at 12 weeks which further improved to 2.2 m/s at 52 weeks (Williams et al., 2017). This is consistent with our HFrEF patients, where there is an average reduction of PWV by 1.98 m/s at 12 weeks with ARNi. In our data, although not statistically different, PWV was numerically higher at baseline in the ARNi group. While 12 weeks of ARNi therapy significantly reduced PWV in HFrEF patients, the PWV values were reduced to similar Control group values at baseline. From a clinical perspective, increases in PWV are associated with an increased risk of CV events (Ben‐Shlomo et al., 2014), and a PWV above 10 m/s is associated with an decreased probability of survival (Hametner et al., 2021). Our ARNi group baseline PWV was 9 m/s; thus it is critical to prevent a further increase in arterial stiffness towards the threshold of 10 m/s to improve outcomes in these patients. Although the ARNi group improved stiffness to match those of the Control group, any level of improvement in this chronic disease population is valuable. Moreover, longer follow‐up may have shown more reduction in PWV as seen in previous trials of ARNi in hypertensive patients when followed over 52 weeks when compared to 12 weeks (Williams et al., 2017).

Several mechanisms may contribute to the improvement in arterial stiffness observed with ARNi. One may be due in part to a reduced BP. Subjects in the ARNi group had higher BP compared to the Control group at baseline. There was a significant reduction in BP after 12 weeks of ARNi therapy, but BP after therapy was similar to Control at baseline. Nevertheless, after statistically accounting for the effect of BP using ANCOVA, the effect of ARNi on arterial stiffness remained. ARNi may also improve central aortic stiffness by increasing endogenous NPs and rebalancing the neurohormonal imbalance. An improvement of the functional component of arterial stiffness may be mediated through NO. Other possible mechanisms for improvement in arterial stiffness may be through anti‐inflammatory properties and sympatho‐inhibitory effects of ARNi. ARNi has been reported to reduce pro‐inflammatory biomarkers, tumor necrosis factor‐alpha (TNF‐α), and interleukin‐18 (IL‐18) in HFrEF (Bunsawat et al., 2020). This, in turn, may reduce elevated oxidative stress seen in HFrEF, reducing vascular inflammation. Moreover, the reduction in sympathetic activity seen with ARNi (Bunsawat et al., 2021), may impact vascular stiffening and elastance. Indeed, it has been shown that heightened sympathetic nerve activity is associated with greater arterial stiffness (Holwerda et al., 2019). Thus, improvements in sympathetic tone and vascular inflammation may be partially responsible for improving arterial stiffness seen with ARNi in HFrEF.

Improved arterial stiffness could be a potential mechanism for the clinical benefits seen with ARNi in patients with HFrEF. In HFrEF, increased arterial stiffness causes early arrival of the reflected wave in the late systolic phase leading to premature closure of the aortic valve, curtailing the stroke volume and cardiac output (Chirinos et al., 2019; Paglia et al., 2014). Increased late systolic LV afterload contributes to LV remodeling with increased myocardial fibrosis (Puntmann et al., 2014) and LV dysfunction progression (Weber & Chirinos, 2018). In addition, there is reduced myocardial oxygen supply due to reduced coronary blood flow because of reduced DBP from the early arrival of the reflected wave in the systolic phase instead of the diastolic phase of the cardiac cycle (Weber & Chirinos, 2018). ARNi, by reducing arterial stiffness, may delay the return of the reflected wave so that it arrives later in the diastolic phase. Thus, this may reduce the LV afterload further reducing cardiac workload, and may also improve myocardial blood flow increasing myocardial oxygen supply. Thus, ARNi may improve LV function by rebalancing the myocardial oxygen supply and demand mismatch.

### 4.2 ARNi and flow‐mediated dilation

This study is also unique in that we demonstrate that ARNi improves endothelial function in patients with HFrEF compared to conventional treatment. This improvement is despite patients in our study being on goal‐directed medical therapy, including maximal vasodilators at baseline. Our findings complement a recent study where 11 HFrEF patients were longitudinally followed and brachial artery FMD was assessed at baseline (before starting ARNi) and at 1, 2, and 3 months after ARNi initiation (Bunsawat et al., 2020). Importantly, our data are consistent with this prior study in showing a ~3% increase in FMD with ARNi therapy, thereby demonstrating strong reproducibility of the effects of ARNi on FMD in HFrEF. In our sample, although not statistically different, FMD was numerically lower at baseline in the ARNi group compared to Control. Therefore, the increase in FMD we observed after 12‐weeks of ARNi matched that of the control group (i.e., similar to control). However, it is important to consider the predictive benefit of measuring changes in FMD (Green et al., 2011). Utilizing the protocol in the current study to perform FMD, prior meta‐analyses demonstrate that for every 1% increase in FMD there is a relative risk reduction of future CVD events by 9% (Green et al., 2011). Thus, despite a small sample size, our data are consistent with other prior data of similar sample size in showing an improvement in FMD with ARNi.

In addition to FMD, the aforementioned study (Bunsawat et al., 2020) also assessed functional capacity with a 6‐min walk test and proinflammatory biomarkers, TNF‐α and IL‐18, were measured. After one month of ARNi therapy, FMD improved, which persisted over the second and third months. There was also an improvement in functional capacity with the reduction in TNF‐α and IL‐18 at 3 months. These preliminary findings provide mechanistic insight into the clinical benefits seen with ARNi in HFrEF. The improvement in FMD was likely due to ARNi; however, this may also reflect vascular function evolution in HFrEF patients on goal‐directed medical therapy. Moreover, there was also a reduction in BP over 3 months in this study, though it was not statistically significant. As BP is an independent determinant of FMD (Maruhashi et al., 2013), there is a possibility of BP impacting FMD. However, we also show that improvement in FMD with ARNi in HFrEF persisted after adjusting for BP, highlighting the additional benefit of ARNi on FMD is beyond its BP‐lowering effects.

Previously, data in animal models of HFrEF have reported improvements in ex‐vivo vascular function in hypertensive rats with surgically induced heart failure with ARNi (Trivedi et al., 2018). Vascular improvements were documented through sustained aortic vasorelaxation responses to acetylcholine and sodium nitroprusside and increased vascular compliance. This improved vascular response was mainly attributable to increased circulating NO, a key mediator in endothelial function. ARNi has also been shown to have reno‐protective benefits in chronic kidney disease animal models (Jing et al., 2017; Suematsu et al., 2018), which is thought to be due to improved NO availability and the possibility of improvement of endothelial function in the kidneys. Our data demonstrated ARNi treatment in HFrEF improves FMD, an assessment of conduit artery endothelial function assessment mediated mainly by NO (Green et al., 2014), and thereby extends the findings seen in animals to humans, further complimenting the recent study seen in humans (Bunsawat et al., 2020).

Neurohormonal imbalance, which is central to the pathophysiology of HFrEF, is mediated mainly by increased endothelin‐1 and reduced NO and causes vascular smooth muscle cell growth, vascular remodeling, endothelial dysfunction, atherosclerosis, and disabling endothelial repair (Marti et al., 2012). Improvement in endothelial function from ARNi could be due to improvements in neurohormonal imbalance by increasing NO bioavailability. ARNi, through neprilysin inhibition, increases endogenous vasodilatory peptides, NPs (Braunwald, 2015), adrenomedullin (Wilkinson et al., 2001), and bradykinin (Cruden et al., 2004). NP after binding to NP receptor, widely expressed in the endothelium, may activate endothelial NO synthase, which increases NO. ARNi also increases other vasodilatory peptides like adrenomedullin and bradykinin, which may also increase NO bioavailability (Cruden et al., 2004; Wilkinson et al., 2001).

### 4.3 ARNi, sedentary time, and KCCQ‐12

Improved LV function and exercise capacity have shown to be favorable prognostic indicators in HFrEF (Cintron et al., 1993; Fleg et al., 2015). Though we did not measure the maximum rate of oxygen consumption or other aerobic fitness measures, we did not observe any changes in sedentary time or physical activity with ARNi treatment. While we recognize this is a small sample and short duration (12 weeks), it appears that vascular function improved in the absence of changes in sedentary time, and therefore cannot be due to changes in activity or sedentary behavior. Although there was no significant improvement in KCCQ‐12 score in the current study, the data were in the hypothesized direction with higher scores after 12 weeks of ARNi. This may be due to the small sample size in this pilot study or the short duration (12 weeks) of observation. Previous studies have shown improvement in physical activity and quality of life after longer follow‐up (Lewis et al., 2017; Malfatto et al., 2020). Nevertheless, we did observe significant improvements in BP (as expected) as well as vascular function, despite no change in physical activity. Lastly, this improvement in vascular function with ARNi may be an initial effect, which in turn may be partially responsible for the long‐term improvement in physical activity and quality of life of ARNi in HFrEF that has been previously reported (Lewis et al., 2017; Malfatto et al., 2020). Therefore, improved vascular function may be a potential mechanism for the clinical benefits seen with ARNi in patients with HFrEF.

### 4.4 Perspectives and significance

Assessment of vascular function in HFrEF is essential as patients with HFrEF with abnormal vascular function have higher morbidity and mortality (Bonapace et al., 2013; Fischer et al., 2005; Meyer et al., 2005; Shechter et al., 2009), and improving endothelial function in HFrEF has also been shown to improve CV outcomes (Hambrecht et al., 1998). We show that ARNi improves vascular function, assessed by PWV and FMD in HFrEF compared to conventional treatment. This may be responsible for improving the quality of life, reducing mortality, and reducing recurrent heart failure admissions. Identifying the pleiotropic effects of ARNi in HFrEF is significant for multiple reasons. First, understanding the physiological mechanism of benefits of ARNi, which is the first in its class, may open new avenues of investigation to identify innovative therapies for HFrEF. Second, this may help identify better risk stratification strategies of early‐stage HFrEF patients based on impaired vascular function and treat them early, thus preventing further deterioration to end‐stage heart failure and/or death. Vascular function assessments may enable better individualization of therapeutic approaches for patients with HFrEF. Third, understanding the mechanisms and additional benefits of this new class of medication will pave the way to evaluate its use in other CV diseases.

### 4.5 Limitations

We recognize our study is not without limitations. Although we have a control group, this study was not a randomized clinical trial. Considering ARNi has received Class I recommendation in HFrEF (Maddox et al., 2021), it would be unethical to conduct a randomized study. The imbalance between groups was reduced by recruiting participants of similar primary characteristics, as demonstrated by similarities in our subject demographics. However, we recognize this study's sample size was small, there were fewer female participants in ARNi group, and participants were not matched on some parameters of baseline vascular function. As such, we cannot rule out that the changes in PWV and FMD observed during the 12‐weeks of ARNi could be due to regression towards the mean as their post‐intervention vascular function was similar to the Control group. Nevertheless, the findings of this pilot study, though they may not be definitive, confirm a recent study showing improvement in vascular function with ARNi in eleven HFrEF participants (Bunsawat et al., 2020). We also did not explore potential mechanisms for what could be driving the changes in vascular function. We had a broad spectrum of participants with HFrEF, including individuals with ischemic and non‐ischemic causes and moderate to severe symptoms. The impact of ARNi may not be uniform in all subgroups, which is an important question to be addressed with a larger trial. HFrEF with severe symptoms (NYHA Class IV) and/or acute decompensation (hypotension and life‐threatening arrhythmias) were excluded from this study; therefore, these results may not apply to individuals with more advanced HFrEF. As we studied only stable HFrEF participants, our conclusions may not be valid in other CV disease populations. Further studies are needed to evaluate the impact of ARNi on other CV diseases and need to examine potential sex differences in response to this type of therapy.

## 5 CONCLUSION

In this pilot study, our data suggest that ARNi may improve vascular function, assessed by arterial stiffness and endothelial function, in patients with HFrEF compared to patients that remained on their conventional treatment. These data add to our understanding of the possible mechanisms that may play a role in the reduced mortality and hospitalizations noted with ARNi in HFrEF. Further studies are needed to evaluate the probable molecular mechanisms involved and evaluate the role of ARNi on arterial stiffness and endothelial function in other CV diseases, including heart failure with preserved ejection fraction, hypertension, and chronic kidney disease.

## CONFLICT OF INTEREST

Dr. Hosmane is a member of the Speaker's Bureau of Novartis Pharmaceuticals. All other authors have no relationships to disclose.

## AUTHOR CONTRIBUTION

S.N and M.M.W. conceived and designed research; S.N., S.M., and M.M.W. performed experiments; S.N and M.M.W. analyzed data and interpreted results of experiments; S.N. prepared figures; S.N., M.A.W., and M.M.W., drafted manuscript; S.N., S.M., M.A.W., W.B.F., D.G.E, and M.M.W. edited and revised manuscript; S.N., S.M., M.A.W., W.B.F., D.G.E, V.H., and M.M.W. approved final version of manuscript.
